# Comparison of ARIMA and Random Forest time series models for prediction of avian influenza H5N1 outbreaks

**DOI:** 10.1186/1471-2105-15-276

**Published:** 2014-08-13

**Authors:** Michael J Kane, Natalie Price, Matthew Scotch, Peter Rabinowitz

**Affiliations:** Yale Center for Analytical Sciences, Yale University, New Haven, CT USA; Biostatistics Department, Yale University, New Haven, CT USA; School of Public Health, Harvard University, Cambridge, MA USA; Department of Biomedical Informatics, College of Health Solutions, Arizona State University, Tempe, AZ USA; Center for Environmental Security, Biodesign Institute and Security and Defense Systems Initiative, Arizona State University, Tempe, AZ USA; Environmental and Occupational Health Sciences, University of Washington, Seattle, WA USA

## Abstract

**Background:**

Time series models can play an important role in disease prediction. Incidence data can be used to predict the future occurrence of disease events. Developments in modeling approaches provide an opportunity to compare different time series models for predictive power.

**Results:**

We applied ARIMA and Random Forest time series models to incidence data of outbreaks of highly pathogenic avian influenza (H5N1) in Egypt, available through the online EMPRES-I system. We found that the Random Forest model outperformed the ARIMA model in predictive ability. Furthermore, we found that the Random Forest model is effective for predicting outbreaks of H5N1 in Egypt.

**Conclusions:**

Random Forest time series modeling provides enhanced predictive ability over existing time series models for the prediction of infectious disease outbreaks. This result, along with those showing the concordance between bird and human outbreaks (Rabinowitz et al. 2012), provides a new approach to predicting these dangerous outbreaks in bird populations based on existing, freely available data. Our analysis uncovers the time-series structure of outbreak severity for highly pathogenic avain influenza (H5N1) in Egypt.

## Background

Certain infectious diseases and disease events occur in a cyclic or rhythmic pattern related to climate or other factors that allows for modeling and prediction of future outbreaks. By understanding when a future outbreak may occur, preventative steps can be taken to minimize its impact. Examples of such preventive steps include vector control, public health messaging to avoid high-risk behaviors or areas, and raising clinician awareness for early diagnosis and treatment. For such prevention to take place, timely and accurate prediction of outbreaks is critical. More than two thirds of emerging infectious diseases in recent decades are zoonotic in origin (crossing from animals to humans) [1, 2]. An example is the recent emergence of highly pathogenic avian influenza. Therefore, an important application of new, improved predictive models should be related to the emergence of zoonotic disease outbreaks in animals that could constitute health risk to nearby human populations.

H5N1 was first detected in poultry populations in Egypt in February of 2006 and was shortly afterward found in 15 governorates around the country. Outbreaks of H5N1 among small farming operations have remained prevalent over time, despite control measures [3]. These outbreaks have caused the death of millions of birds due to illness or due to culling to prevent further spread of the disease. In addition to bird cases, 168 humans in Egypt have been confirmed as infected with H5N1 as of August, 2012, and 60 of those cases resulted in death due to the disease [4]. Along with being a menace to human health, the continued presence of the pathogen in Egypt impacts the country’s protein sources, farmer’s livelihoods, the economy as a whole. Furthermore, continued transmission of the pathogen between birds (and from birds) to humans has the potential to drive virus evolution with the possibility of increased virulence, and human-to-human transmission. These events may increase the likelihood global pandemic among humans [5].

In Egypt, it has been reported that H5N1 outbreaks in bird populations occur in close temporal proximity to human H5N1 cases, with human cases generally occurring within 30 days of an initial bird outbreak. It has also been reported that daily temperature could affect the risk of disease transmission [6]. As a result, by tracking and predicting the number of bird outbreaks we hope to predict increases in risk for the zoonotic (animal to human) transmission of the virus. Therefore, development of better prediction models for avian influenza outbreaks in Egypt could aid efforts to control and prevent disease in both birds and humans.

For diseases that occur in cyclic or repeating patterns, time series models have been used to predict future outbreaks. Traditionally, time series predictions are performed using the autoregressive integrated moving average (ARIMA) models, which attempt to filter out high-frequency noise in the data to detect local trends based on linear dependence in observations in the series. These models have two distinct advantages. First, they can be easily interpreted in retrospective studies. Like ordinary least-square regressions the relationship between the independent variables and the dependent variables are easily understood based on the assumptions of the model. This allows a user to understand not only the relationship between the current state as a function of the past states, commonly referred to as endogenous variables, but also the influence of inputs outside the state of the series, also called exogenous variables. The second advantage of ARIMA models is that model selection can be performed over a time series in an automated fashion to maximize prediction accuracy.

For some systems the relationships governing dynamic behavior change over time. As a result, prediction parameters in the model must be recalibrated to accurately predict future observations in the face of these changing relationships. The ARIMA model accommodates these dynamic relationships, updating the model based on recent events to predict the future state of the system.

The ARIMA model was originally conceived for economics applications but has seen widespread use in the area of infectious disease for a number of different time varying events. These include leptospirosis and its relationship to rainfall and temperature [7], the role of El Niño southern oscillation (ENSO) climate events on malaria case incidence [8], and the relationship between changes in national alcohol policies and suicide incidence [9]. ARIMA models have also been used in a number of influenza studies. These studies include associating climatological factors with influenza outbreaks in two different climates [10], and to compare all-cause mortality with influenza epidemics [11]. Surprisingly, for reasons that are not clear, they have not previously been applied to challenge of predicting recurrent avian influenza outbreaks in poultry.

Although it has become a standard tool for time series, the ARIMA model does suffer from two drawbacks. First, it assumes linear relationships between independent and dependent variables. Real-world relationships are often non-linear and therefore more complex than the assumptions built into the model. As a result the ARIMA model often does not perform well where data has structure but the structure is complex. Second, the ARIMA model assumes a constant standard deviation in errors in the model over time. This assumption can be removed when the ARIMA is used in conjunction with a Generalized AutoRegressive Conditional Heteroskedasticity (GARCH) model [12], which attempts to characterize a model’s heteroskedasticity or non-constant standard deviations in a time series. However, the GARCH model comes with its own challenges and optimizing the GARCH model parameters for a time series can be problematic.

More recently a new class of regression models have been developed to address the challenges associated with the classic models. This paper makes use of Random Forest regression [13], which starts by creating decision trees. Decision trees recursively partition data in the regression space until the amount of variation in the subspace is small. A predictor for the subspace can then be created simply by taking the average value of the dependent data corresponding to the independent data in the subspace. The recursive partitioning step can be visualized as a tree; hence the name. Predictions for new data are obtained by finding the predictor corresponding the partition where the new input variable resides.

The partition process for Random Forest is greedy and, as a result, does not generally converge to the globally optimal tree. To compensate for this a collection or ensemble of locally optimal trees, each tree is generated by sampling uniformly at random from the original subset, a procedure termed bagging. Furthermore, after creating the resampled version of the data set, all but a small number of features are sampled. With the sampling procedure complete a new tree is trained. The collection or ensemble of trees is termed a forest. Predictions can then be made based on an aggregate of the individual predictions made by each of the trees, a procedure termed “voting”. An aggregate prediction obtains better accuracy than any of the constituent trees. Random Forests in particular and the multiple-predictor approach in general, also referred to as ensemble methods, tend to be the most accurate classification and regression tools currently at the disposal of data scientists [13].

While Random Forest models often provide superior prediction accuracy, they are often challenging to interpret, with [14] referring to to “algorithmic models”, including Random Forests as a “black box”. For Random Forests specifically, each of the constituent trees trains on a potentially non-linear regression space and is then combined with others. As a result they do not encode the simple and easy-to-understand results provided by classical linear models. Diagnostics for random forests models tend to focus on variable importance. This technique removes regressor variables from an ensemble and measures the effect on prediction accuracy. This approach provides a functional measure of the influence that each variable has on accuracy without providing an interpretable measure of how the variable helps to determine the prediction.

The Random Forest approach has been used in a number of public health studies such as to predict deer mouse population dynamics [15] and the effect of seasonality on wastewater quality [16]. In the area of predicting avian influenza, Random Forests have been successfully applied to spatial challenges such as [17], which provides the first global-scale model of low-pathogenicity avian influenza in wild birds. As with the ARIMA model though, this approach has not been applied to time-series data for predicting avian influenza outbreaks. In this paper, we compared the performance of an ARIMA model with the Random Forest model in the prediction of outbreaks of avian influenza H5N1 in Egypt over the period of 2005–2011 in order to identify optimal qualities of a prediction model for use by public health and agricultural agencies in the control and prevention of future outbreaks in poultry.

## Methods

### Weather and bird outbreak data acquisition

Avian influenza outbreak data were obtained from the online EMPRES-i Global Animal Disease Information System [18]. EMPRES records contain verified reports of viral infection in animal populations including data on avian influenza H5N1. Individual cases of H5N1 are confirmed by the Food and Agricultural Organization of the United Nations. For this study, an “outbreak” is defined by at least a single, confirmed case of H5N1 in a domestic bird (chicken, duck, goose, turkey, or unspecified bird type) at a given location. It should be noted that this means an outbreak at a large facility has the same significance or weight as a case at a small one. This decision was justified for two reasons. First, while the confirmation of a case of avian influenza is carefully recorded the total number of cases at a location is not. For some locations, the number of cases was the same for multiple outbreaks indicating either that the same number of birds were confirmed to have H5N1 or there was a recording error. As a result, the data may do not reflect the actual number of cases at a given location. Second, in Egypt, birds are often cultivated in small, unregulated farms and enclosures. It is conceivable, if not likely, that outbreaks at these small facilities are underreported. By defining an outbreak as at least a single, confirmed case our analysis is not sensitive to case-count discrepancies presented by the first challenge. At the same time, we are placing more emphasis on underreported facilities thereby addressing the second challenge. The new “outbreak” variable was constructed from the EMPRESS data and is used throughout this study.

Daily temperature and relative humidity data were obtained from the Weather Underground [19]. The site provides publicly available historical data from weather stations around the world. We identified weather stations located in each governorate in Egypt with avian influenza cases that had consistent daily readings, from 2005-12-08 to the 2012-10-28.

The data were merged into a single set with variables corresponding to date of the beginning of a week, number of bird outbreaks, average temperature for all governorates, and humidity.

### Retrospective ARIMA and Random Forest model

A retrospective analysis was performed on the data using both an ARIMA model as well as a Random Forests. In both cases the entire data set, and all associated variables were used to train the model. The ARIMA model was selected using the forecast package [20], which is available for the R programming environment [21]. Model selection was automated, using the auto.arima function, which performs a stepwise regression on the data and selects the best model based on the Bayesian Inference Criteria (BIC). The order of the autoregressive, integral, and moving average parameters of the model were limited to five or less to make the computation tractable.

The Random Forest model was created using the randomForest package [22], also available for the R programming environment. As with the ARIMA model, all variables were included and time lags of up to five steps were trained. The Random Forests model used 1000 trees, sampling all rows of the data set with replacement, and sampling 3 variables at random in each of the trees. The importance of each variable was then calculated by finding how much of a reduction each variable provides when added to the model.

### Simulated prospective ARIMA and Random Forest model

The retrospective analysis establishes the relationships between outbreaks and the regressor variables for 2007 to the present. However, it is well known that models based on retrospective relationships do not always imply good predictive models. To investigate the predictive power of the ARIMA and the Random Forest models, a simulated prospective analysis was performed. The simulation begins by using data from the last 30 weeks of data from 2006 to predict the outbreak for the week of 2007 (2007-01-07). The simulation proceeds by iteratively adding a new week of data, training a new model based on the updated data, and predicting the number of outbreaks for the following week. The procedure for creating the ARIMA and Random Forest model was otherwise the same as in the retrospective analysis.

### Validating the prospective Random Forest model

The results from the simulated prospective analyses showed that the Random Forest model is a more accurate predictor of future outbreaks, in terms of mean square error. Two procedures were used to show that the Random Forest model additionally provides predictive power, thereby validating the model. First, a confusion matrix was constructed with summarizing the model ability to predict upticks and downticks for each week. A test was then constructed to see if the predicted upticks happen at the same rate as if up and downticks were random distributed with the same probability as the proportion of up and downticks in the actual outbreaks. This test verifies that the random forest model can predict the direction of change in actual outbreak. To show that the magnitudes of the predictions predict actual outbreak magnitude, the predictions were examined graphically and a quantile plot was constructed to better understand the distribution of the residuals.

## Results

### Retrospective ARIMA

Table 1 shows the summary of the retrospective ARIMA model. Using the optimization described earlier, the autoregressive component of the model was of order 1, the integrated portion of the model was of order zero, and the moving average component was of order 2. The table also includes the p-values for the coefficients using the standard t-test. The variance of the residuals was 26.9697. It should be noted that the effect of temperature and humidity were studied similarly but did not yield significant results. Temperature and humidity each had a small (not significant) negative effect when added to the model both individually and together.

**Figure 1:**
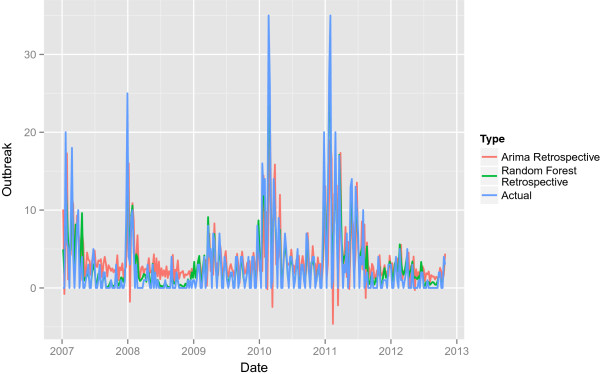
**The retrospective predictions.** The retrospective predictions for the ARIMA model and the Random Forest model along with the actual outbreak counts.

**Figure 2:**
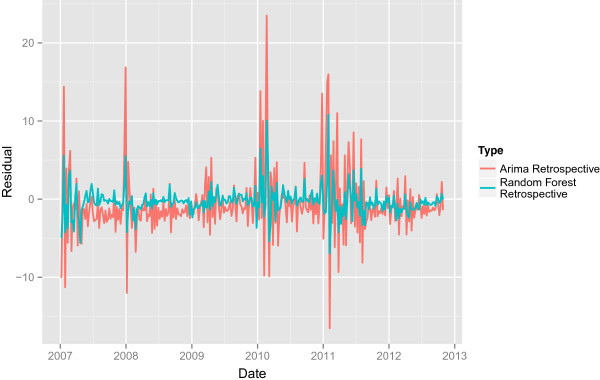
**The retrospective residuals.** The retrospective residuals for the ARIMA and Random Forest model.

Figures 1 and 2 give graphical representation of the retrospective ARIMA and Random Forests model. The figures show that there tend to be sharp increases in outbreak frequency in the winter months and that the residuals corresponding to these drastic upticks tend to be larger. Furthermore, 2010–2012 tended to have higher outbreak counts along with more drastic up and downticks through the entire period. The corresponding residuals over this period tend to be larger.

**Table 1:** **Summary of the retrospective ARIMA model**

	Autoregressive 1	Moving average 1	Moving average 2	Intercept
Coefficient estimate	0.8367	-0.2841	-0.2469	6.2052
Standard error	0.0700	0.0915	0.0647	1.5203
p-value	0.0014	0.0054	0.0044	0.0041

### Retrospective Random Forest

Table 2 shows the variable importance for each of the regressors in the Random Forest model as defined in [13]. Figures 1 and 2 also show the performance for the retrospective Random Forest model. As in the case of the ARIMA, the Random Forest model has trouble predicting sharp increases and decreases in outbreaks and has more trouble predicting the time series over the volatile period of 2010–2012. However, the overall variance for the retrospective Random Forest model is considerable smaller with a mean square error of 6.3195 compared to 26.9597 for the ARIMA model.

**Table 2:** **Retrospective random forest variable importance**

Variable name	Percent increase in MSE
Outbreak Lag 1	0.9132981
Outbreak lag 2	3.753933
Outbreak lag 3	10.2857484
Temp lag 1	3.6919581
Temp Lag 2	3.5478696
Temp lag 3	4.3230635
Humidity lag 1	3.6816384
Humidity lag 2	6.4406314
Humidity lag 3	3.5846449

### Prospective ARIMA and Random Forest

**Figure 3:**
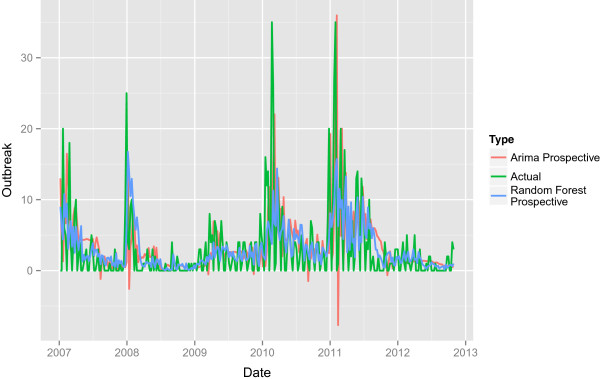
**The simulated prospective predictions.** The simulated prospective predictions for the ARIMA model and the Random Forest model along with the actual outbreak counts.

**Figure 4:**
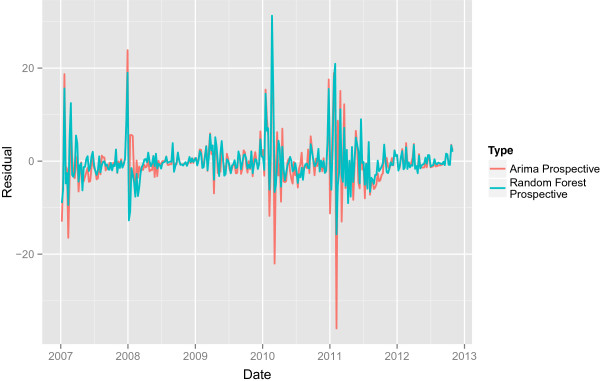
**The simulated prospective residuals.** The simulated prospective residuals for the ARIMA and Random Forest model.

Figures 3 and 4 and show the performance of the prospective ARIMA and Random Forests models respectively. As with the retrospective models, the high frequency shocks that correspond to steep increases in outbreak near the beginning of the year are difficult to predict. The period of 2010–2012 is associated with higher volatility in the residuals. As in the retrospective case, the predictive ARIMA makes predictions less than zero. The simulated prospective models also do not tend to make predictions for large magnitude changes. Finally, the Random Forest model’s MSE, at 24.8101 is smaller than the ARIMA’s simulated prospective MSE (28.7412) as well as the retrospective ARIMA’s MSE (26.9597).

### Comparing the MSE of the models

Table 3 shows the mean square error of the residuals for each of the four models for the prediction period of 2007-01-07 to 2012-10-28. The retrospective models perform better than their simulated prospective counter parts and the Random Forest simulated prospective model outperforms both the retrospective and simulated prospective ARIMA model.

**Table 3:** **Comparing the MSE of the models**

	Retrospective	Prospective
ARIMA	26.9597	28.7412
Random Forest	6.3195	24.8101

### Validating the prospective Random Forest model

The prospective Random Forest model outperforms the ARIMA over the specified time period, measured by means square error. However, this fact alone does not guarantee that the Random Forest model is able to anticipate upticks or downticks in outbreak. To show that it does have this capability, a confusion matrix was created that shows the proportions of predicted increases and decreases in outbreak with actual increases and decreases in outbreak.

From the data we know that the proportion of actual increases was 0.5908 and the proportion of predicted increases was 0.6238. If we assume that, as a null hypothesis, that the predicted and actual increases and decreases happen independently of each other, then under the null we would have the confusion matrix shown in Table 4.

**Table 4:** **The confusion matrix under the null**

	Predicted		
		Up	Down
**Actual**	Up	0.3685	0.2222
	Down	0.2553	0.154

A *χ*^2^ test was performed to find the probability that the confusion matrix in Table 5 could be generated under the null in Table 5 and the result was numerically zero. We can therefore conclude that the prospective Random Forest model has the ability to predict increases and decreases in outbreaks.

**Figure 5:**
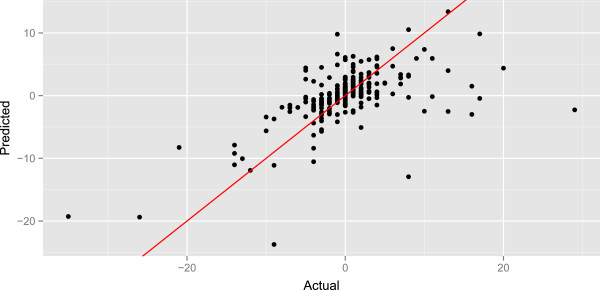
**The actual and predicted changes for the Random Forest model.** The actual and predicted changes in outbreak for each day of the simulated prospective study using the Random Forest model.

Figure 5 shows the actual and predicted changes in outbreak for each day of the simulated prospective study. Points in the first and third quadrant correspond to cases where the direction of a prediction coincided with the actual outbreak change. Points near the diagonal red line correspond to predictions that were accurate.

**Figure 6:**
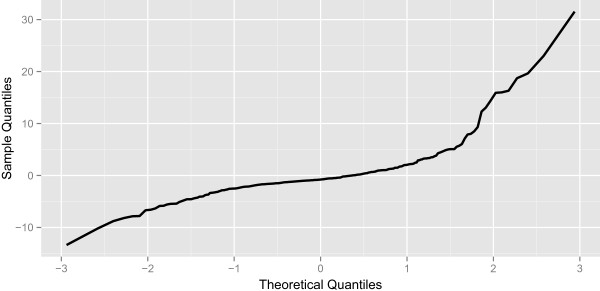
**Quantile plot of the residuals for the simulated prospective analysis using Random Forests.** The normal quantile plot of the differences between the predicted changes and the actual changes using the Random Forest model.

Figure 6 shows the normal quantile plot of the residuals between the predicted and actual changes. The plot is roughly linear from -3 to approximately 0.75, corresponding to a normal distribution. From 0.75 to 3 the plot curves upward, corresponding to the steep increases in outbreaks that were not well predicted. Based on this information we can surmise that the noise process is skewed right.

**Table 5:** **Simulated prospective Random Forest confusion matrix**

	Predicted		
		Up	Down
**Actual**	Up	0.5083	0.0825
	Down	0.1155	0.2937

## Discussion

These analyses indicate that the Random Forest model has advantages over the ARIMA approach to time series modeling of avian influenza outbreaks in poultry in Egypt. At the same time, it clear that both retrospective models have deficiencies when trying to fit the time series of outbreaks. For example, the ARIMA model provides some estimates that are actually less than zero, which is impossible given the nature of outbreaks. Furthermore, there are times, like the end of 2008 where the model is consistently biased with respect to the signal. The Random Forest model is also consistently biased at the end of 2008, as well as the middle of 2012. However, it performs an order of magnitude better than ARIMA in terms of mean square error.

These periods where both retrospective models are biased in their predictions may indicate that the distribution of the noise process is itself changing over the course of the almost five years that were examined. There are many possible factors that could influence the distribution such as changes in the data collection procedure, changes in regulation related to the handling of outbreaks, or changes in disease dynamics. However, this clearly needs to be taken into account when proposing a predictive model.

The choice to use 25-week windows when performing predictions was an effective means for handling distribution changes in the prospective setting. Assuming that the distribution changes occur slowly over time allows us to assume quasi-stationarity of the process and the models can be trained to filter out this type of noise much more easily. As a result of this windowing procedure the prospective ARIMA’s performance is competitive with the retrospective ARIMA. The prospective Random Forest model performed better than both ARIMA models making it the best choice for-out-of sample prediction.

The prospective Random Forest model likely outperformed the prospective ARIMA model for two distinct reasons. First, the relationship between the lagged outbreaks and the predicted outbreak may not be linear. From Table 2, where the importance for each of the variable used by the Random Forest model is shown we know that the 3 week lagged outbreak is one of the most important variables used by the model to predict outbreaks. However no corresponding term appears in the ARIMA model, which assumes linear relationships between the predicted outbreak and lagged outbreak values. The ARIMA’s inability to incorporate non-linear relationships may have contributed to poorer performance. Second, from Figure 6 we know that upward shocks in outbreak are not well predicted and, as a result, the noise process of the time series is not normal, which is one of the basic assumptions made by the ARIMA model. This fact may have also contributed to poorer performance.

## Conclusions

The Random Forest approach offers advantages over the ARIMA approach for prediction of H5N1 avian outbreaks in birds in Egypt. Further research is warranted to explore the utility of such novel time series models in other settings, including as part of a concerted effort by agricultural and public health agencies to control and prevent this disease.
